# Identification and validation of the diagnostic signature associated with immune microenvironment of acute kidney injury based on ferroptosis-related genes through integrated bioinformatics analysis and machine learning

**DOI:** 10.3389/fcell.2023.1210714

**Published:** 2023-07-27

**Authors:** Yalei Chen, Anqi Liu, Hunan Liu, Guangyan Cai, Nianfang Lu, Jianwen Chen

**Affiliations:** ^1^ Department of Critical Care Medicine, Capital Medical University Electric Power Teaching Hospital/State Grid Beijing Electric Power Hospital, Beijing, China; ^2^ State Key Laboratory of Kidney Diseases, Beijing Key Laboratory of Kidney Disease Research, Department of Nephrology, First Medical Center of Chinese PLA General Hospital, National Clinical Research Center for Kidney Diseases, Nephrology Institute of the Chinese People’s Liberation Army, Beijing, China

**Keywords:** Acute kidney injury, ferroptosis-related genes, immune microenvironment, machine learning, diagnostic signature

## Abstract

**Background:** Acute kidney injury (AKI) is a common and severe disease, which poses a global health burden with high morbidity and mortality. In recent years, ferroptosis has been recognized as being deeply related to Acute kidney injury. Our aim is to develop a diagnostic signature for Acute kidney injury based on ferroptosis-related genes (FRGs) through integrated bioinformatics analysis and machine learning.

**Methods:** Our previously uploaded mouse Acute kidney injury dataset GSE192883 and another dataset, GSE153625, were downloaded to identify commonly expressed differentially expressed genes (coDEGs) through bioinformatic analysis. The FRGs were then overlapped with the coDEGs to identify differentially expressed FRGs (deFRGs). Immune cell infiltration was used to investigate immune cell dysregulation in Acute kidney injury. Functional enrichment analysis and protein-protein interaction network analysis were applied to identify candidate hub genes for Acute kidney injury. Then, receiver operator characteristic curve analysis and machine learning analysis (Lasso) were used to screen for diagnostic markers in two human datasets. Finally, these potential biomarkers were validated by quantitative real-time PCR in an Acute kidney injury model and across multiple datasets.

**Results:** A total of 885 coDEGs and 33 deFRGs were commonly identified as differentially expressed in both GSE192883 and GSE153625 datasets. In cluster 1 of the coDEGs PPI network, we found a group of 20 genes clustered together with deFRGs, resulting in a total of 48 upregulated hub genes being identified. After ROC analysis, we discovered that 25 hub genes had an area under the curve (AUC) greater than 0.7; Lcn2, Plin2, and Atf3 all had AUCs over than this threshold in both human datasets GSE217427 and GSE139061. Through Lasso analysis, four hub genes (Lcn2, Atf3, Pir, and Mcm3) were screened for building a nomogram and evaluating diagnostic value. Finally, the expression of these four genes was validated in Acute kidney injury datasets and laboratory investigations, revealing that they may serve as ideal ferroptosis markers for Acute kidney injury.

**Conclusion:** Four hub genes (Lcn2, Atf3, Pir, and Mcm3) were identified. After verification, the signature’s versatility was confirmed and a nomogram model based on these four genes effectively distinguished Acute kidney injury samples. Our findings provide critical insight into the progression of Acute kidney injury and can guide individualized diagnosis and treatment.

## Introduction

Acute kidney injury (AKI) is a common and severe disease that is associated with a high risk of developing chronic kidney disease (CKD) and end-stage renal disease (ESRD) (Bellomo et al., 2012). The incidence of hospital-acquired AKI is approximately up to 20%, while in the intensive care unit it can be as high as 45% (Kam Tao Li et al., 2013; Chen et al., 2022). AKI is believed to contribute to approximately 1.7 million deaths annually and is a global health burden with high morbidity and mortality (Mehta et al., 2015). Despite extensive investigation into AKI, therapeutic options remain limited, and the underlying mechanisms of AKI are largely unclear (Chen et al., 2020). Therefore, identifying new biomarkers for kidney dysfunction before the onset of AKI may aid in earlier detection and be critical in developing new treatments (Huang et al., 2022).

The main pathology of AKI is the death of renal tubular epithelial cells. Besides apoptosis, other forms of regulated cell death such as ferroptosis and pyroptosis have also been increasingly recognized in recent years (Linkermann et al., 2014). Ferroptosis is a type of iron-dependent regulated necrosis that features intracellular iron retention, depletion of reduced glutathione (GSH), and accumulation of lipid reactive oxygen species (ROS) dependent on iron levels within the cell itself (Martin-Sanchez et al., 2017). Excessive accumulation of ROS activates intracellular oxidative stress, resulting in damage to proteins, nucleic acids, lipids, and ultimately resulting in the occurrence of ferroptosis (Feng et al., 2022). In cells undergoing ferroptosis, shrinking mitochondria are observed, which leads to increased density of the mitochondrial membrane, rupture or vanishing of mitochondrial cristae, and a ruptured outer membrane, whereas the morphology of the nucleus is normal, and the cell membrane remains intact (Hosohata et al., 2022). Recently, ferroptosis has been reported to be involved in AKI (Martin-Sanchez et al., 2017; Feng et al., 2022), and several interventions have been designed to block different nodes of the ferroptosis network, including antioxidants, lipid peroxidation blockade, and iron chelators (Mishima et al., 2020; Jiang et al., 2021; Xiao et al., 2021; Hosohata et al., 2022). It has been reported that augmenter of liver regeneration could regulate the development of ferroptosis through GSH/GPX4; ACSL4 knockout significantly inhibited the ferroptosis of renal tubular epithelial cells in AKI mice; and XJB-5-131, a new generation of antioxidant, could specifically inhibit ferroptosis by inhibiting lipid peroxidation and then alleviate AKI (Zhao et al., 2020; Feng et al., 2022; Wang et al., 2022). These results suggested that ferroptosis is deeply related to AKI, and to find the key ferroptosis-related genes (FRGs) and explore the mechanism of ferroptosis is of great significance for the development of effective treatment strategies for AKI.

In the present study, we aimed to identify novel diagnostic ferroptosis-related genes (FRGs) for AKI based on bioinformatics and machine learning. We analyzed our previously uploaded dataset GSE192883 (Chen et al., 2022) and another mouse dataset GSE153625 (Kim et al., 2020) from Gene Expression Omnibus (GEO) database to determine common differentially expressed genes (DEGs) and hub FRGs between AKI and Control specimens. Then, we analyzed their diagnostic value in AKI using machine learning and receiver operator characteristic (ROC) curve analysis. Finally, we confirmed our findings based on GEO datasets using quantitative real-time PCR (qPCR) in our cohort. Our findings provide novel critical genes involved in the progression of AKI, which can guide individualized diagnosis and treatment.

## Materials and methods

### Data collection

The raw datasets, which include gene expression data for AKI and Control, were downloaded from the GEO database (https://www.ncbi.nlm.nih.gov/geo/). Detailed information was presented in Table 1. In particular, samples in the GSE192883 dataset were classified into several groups based on ischemia time. For this study, we classified the ischemia reperfusion injury (IRI) 28 min and IRI 30min groups as group AKI. Only cortex samples were used in the GSE217427 dataset.

**TABLE 1:** The information of high throughput sequencing datasets obtained from the GEO database.

Dataset	Organism	Year	AKI sample	Control sample	Platform
GSE192883	*Mus musculus*	2022	6	3	GPL28457
GSE153625	*Mus musculus*	2020	4	8	GPL21103
GSE217427	*Homo sapiens*	2022	11	11	GPL24676
GSE139061	*Homo sapiens*	2019	39	9	GPL20301
GSE98622	*Mus musculus*	2017	9	31	GPL13112

GEO, gene expression omnibus; GSE, gene expression omnibus series; AKI, acute kidney injury.

### Identification of DEGs

The linear model for high-throughput data analysis (limma) (Ritchie et al., 2015) in Bioconductor was applied to find DEGs by comparing expression value between AKI samples and Control samples in GSE192883 and GSE153625. Differential expression was calculated using an empirical Bayes model. The criteria for the statistically significant difference of DEGs was | log2 fold change (FC)| ≥ 1 in expression and adjusted *p*-value (false discovery rate, FDR) < 0.05. Volcano plot of all DEGs was performed by ggplot2 package (Ginestet, 2011) in R.

### Weighted gene co-expression network analysis (WGCNA)

WGCNA was adopted to explore the correlation between genes (Zhou et al., 2022) and identify important module genes in AKI. Firstly, the median absolute deviation (MAD) of each gene was determined, and top 5000 genes with the biggest MAD were included for next step. Secondly, the DEG expression matrix was filtered by the goodSamplesGenes function to omit unqualified genes and samples, and a scale-free co-expression network was built. Thirdly, adjacency was computed using the co-expression similarity-derived “soft” thresholding power (β). The adjacency was then converted into a topological overlap matrix (TOM), and the gene ratio and dissimilarity were determined. The fourth step was the detection of modules using hierarchical clustering and a dynamic tree cut function. Genes with identical expression profiles were classified into gene modules using average linkage hierarchical clustering, with a TOM-based dissimilarity metric and a minimum gene group size (n = 50) for the gene dendrogram. Fifthly, the dissimilarity of module eigengenes was computed, a cut line for the module dendrogram was chosen, and several modules were combined for further investigation. The eigengene network was finally visualized.

### Assessment of immune cell infiltration

The CIBERSORT (Newman et al., 2015), a method using the principle of linear support vector regression to deconvolute the expression matrix of 22 immune cell subtypes, was used to explore the discrepancy in immune cell between AKI and Control samples (Fan et al., 2022). Subsequently, we screened out the immune cells that showed significant differences in infiltration between groups.

### Functional enrichment analysis

The “clusterprofiler” R package (Yu et al., 2012) was used to perform Gene Ontology (GO) and Kyoto Encyclopedia of Genes and Genomes (KEGG) functional enrichment analyses (Yu et al., 2012). In the GO enrichment analysis, the categories include the cellular component (CC), the biological process (BP), and the molecular function (MF) terms, and adjusted *p* < 0.05 was regarded as statistically significant differences. In the KEGG pathway enrichment analysis, enriched pathways were identified with an adjusted *p* < 0.05 (J. Chen et al., 2020).

### Protein-protein interaction (PPI) network construction and analysis of modules

Considering that proteins rarely work alone, it is necessary to study the interactions among proteins. The Search Tool for the Retrieval of Interacting Genes/Proteins (STRING) is an online biological resource database (https://cn.string-db.org/) that is commonly used to identify the interactions between known and predicted proteins. By searching the STRING database, the PPI network were selected with a score >0.7, and the PPI network was visualized by Cytoscape software. Finally, the hub genes were screened from this PPI network by Molecular Complex Detection (MCODE) (Bader and Hogue, 2003) and Cyto-Hubba apps (Chin et al., 2014; Chen et al., 2020).

### Identification of differentially expressed FRGs (deFRGs)

FerrDb V2 is the world’s first database (http://www.zhounan.org/ferrdb/current/) that dedicates to ferroptosis regulators and ferroptosis-disease associations (Zhou et al., 2023). A total of 484 regulatory factors including drivers, suppressors and markers were downloaded from FerrDB V2 database. The commonly expressed DEGs (coDEGs) in GSE192883 and GSE153625 were intersected with the genes obtained from FerrDB V2 to obtain deFRGs. Furthermore, these 33 deFRGs were performed enrichment analysis and PPI network constructions.

### Hub gene selection based on machine learning algorithms

Machine learning–based algorithms have been widely used in clinical decision making. Of them, the least absolute shrinkage and selection operator (Lasso) is one of the most commonly used algorithms and its clinical efficacy has been demonstrated previously (Huang et al., 2016; Reichling et al., 2020). Therefore, we choose Lasso model to select the gene signatures associated with AKI. The Lasso model is a dimensionality reduction method for evaluating high dimensional data and was fitted using the “cv.glmnet” function in the R package “glmnet” (Friedman et al., 2010). Firstly, the ROC curves and the area under the curve (AUC) were used to evaluate the diagnostic efficacy of 48 upregulated hub genes in two human datasets (GSE217427 and GSE139061). And then, Lasso model was applied to analysis the 25 hub genes with AUC over than 0.7 in either human dataset to obtain the final AKI-related hub genes.

### Construction of the nomogram model

We created a nomogram model to predict AKI using the R package “rms” (Liu et al., 2021). The diagnostic nomogram model of final 4 hub genes Mcm3, Pir, Atf3, and Lcn2 was constructive in GSE139061 human dataset. The expression of each gene has a corresponding point. The “Total Points” reflected the sum of all the above elements. The ROC curve of the diagnostic nomogram model was performed in GSE139061 human dataset.

### Animals and procedures

C57BL/6 mice (20–25 g) were purchased from the Animal Center of Chinese PLA General Hospital. All animal procedures were approved by the Institutional Animal Care and Use Committee at the Chinese PLA General Hospital and Military Medical College. The 12 male mice were randomly assigned to three groups: Sham group (4 mice underwent sham surgery), bIRI-1d group (4 mice underwent bilateral renal ischemia for 30 min and reperfusion for 1 day), bIRI-7d group (4 mice underwent bilateral renal ischemia for 30 min and reperfusion for 7 days). Renal ischemia and reperfusion and renal sham surgery were performed as described previously (Chen et al., 2022), blood and kidney samples were harvested for further processing.

### Histopathological examination and assessment of kidney injury

A quarter of the kidney was fixed in 4% formaldehyde, dehydrated, and embedded in paraffin. Tissue sections (4 mm) were stained with periodic acid–Schiff (PAS). Kidney injury was assessed by measuring the levels of serum creatinine (SCr) and blood urea nitrogen (BUN). Blood samples were collected from the vena cava at the indicated time points, and the serum was separated by centrifugation at 3,000 rpm for 15 min at 4°C and then sent to the PLA General Hospital Biochemistry Department to detect SCr and BUN.

### Quantitative real-time PCR (qPCR)

Frozen tissue samples were lysed in TRIzol reagent (Invitrogen, Carlsbad, CA, United States), and total RNA was extracted according to the manufacturer’s instructions. The levels of transcripts were determined by qPCR using TransStartTM Top Green qPCR SuperMix (AQ131, Transgen, Beijing, China) on an Applied Biosystems 7500 system PCR cycler (Applied Biosystems, Foster City, CA, United States). The data were normalized to 18S expression and further normalized to the Control group. Primers were obtained from Genomics (BGI Tech, China). All of these primers are listed in Table 2 **.**

**TABLE 2:** Gene specific primers used in our study.

Gene	Forward primer (5′to 3′)	Reverse primer (5′to 3′)	Product length
Names			
18s	GTA​ACC​CGT​TGA​ACC​CCA​TT	CCA​TCC​AAC​GGT​AGT​AGC​G	150bp
Lcn2	TTT​GTT​CCA​AGC​TCC​AGG​GC	ACT​GGT​TGT​AGT​CCG​TGG​TG	106bp
Atf3	AAA​TTG​CTG​CTG​CCA​AGT​GTC	CGG​TGT​CCG​TCC​ATT​CTG​A	200bp
Pir	AGT​CGA​AGG​TTT​ACA​CTC​GCA	AGG​ACT​GCT​GTG​TGA​TGT​GG	181bp
Mcm3	CCA​ATC​CAG​TCT​ATG​GCA​GGT	CCC​TGT​ATT​GGT​GCA​TCC​TCA	171bp

## Results

### Screening of genes associated with AKI in GSE192883 dataset

**FIGURE 1:**
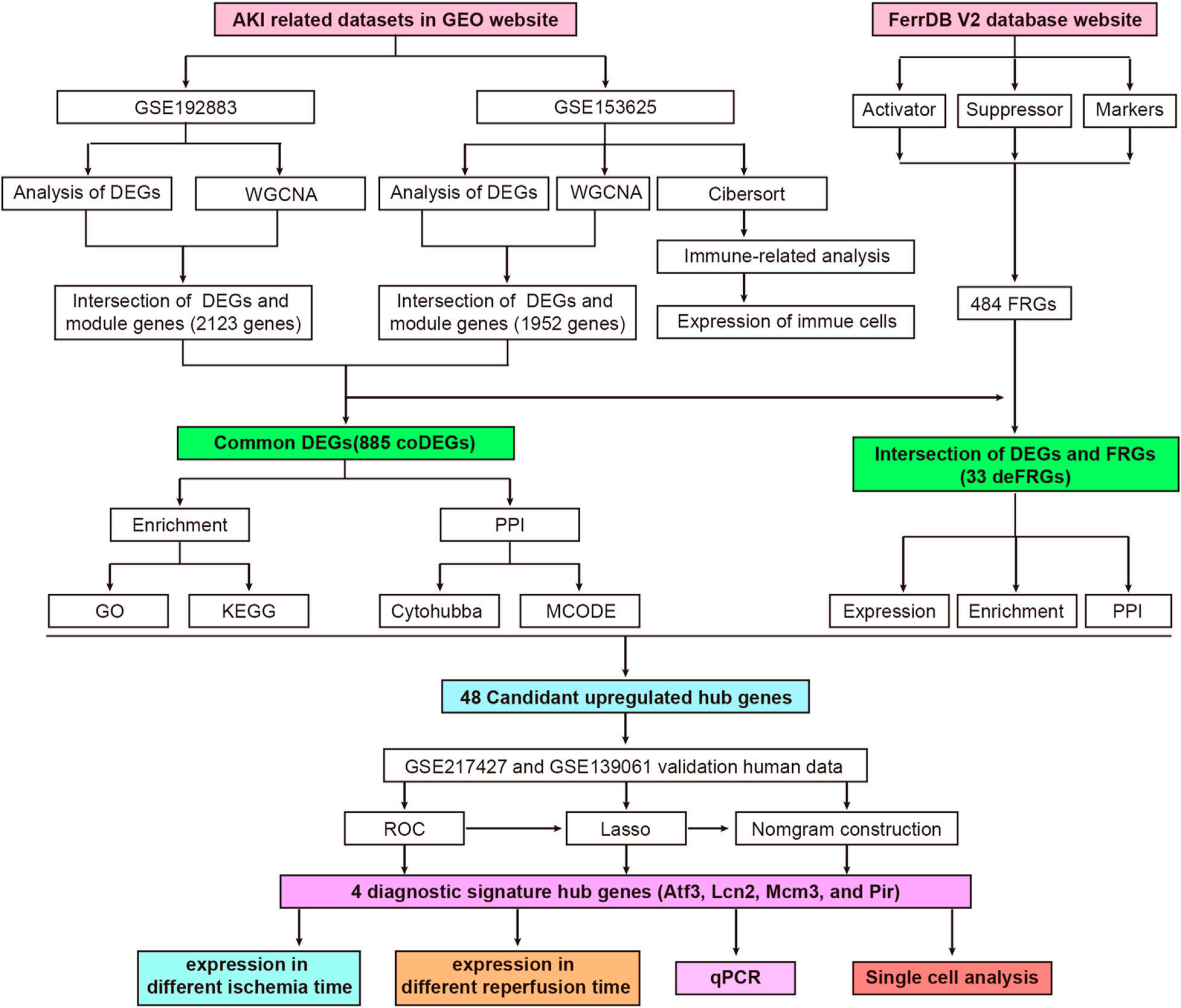
Flowchart of research design and analyzing process of this study. AKI, acute kidney injury; GEO, gene expression omnibus; GSE, gene expression omnibus series; DEGs, differentially expressed genes; WGCNA, weighted gene co-expression network analysis; coDEGs, common DEGs; FRGs, ferroptosis-related genes; deFRGs, differentially expressed FRGs; PPI, protein–protein interaction; GO, gene ontology; KEGG, Kyoto Encyclopedia of Genes and Genomes; MCODE, molecular complex detection; ROC, receiver operating characteristic; Lasso, least absolute shrinkage and selection operator; qPCR, quantitative real-time polymerase chain reaction.

**FIGURE 2:**
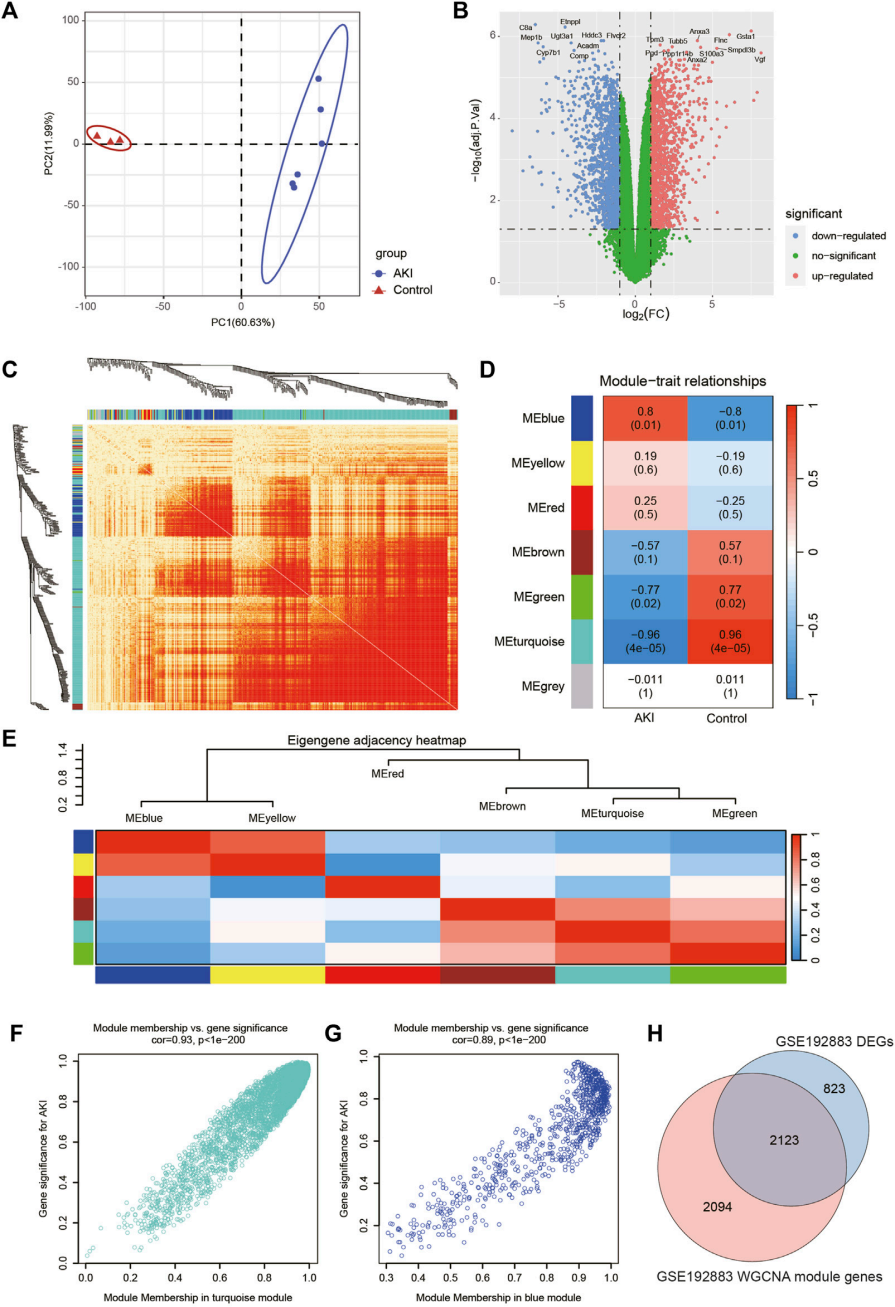
Screening of genes associated with AKI in GSE192883 dataset. **(A)** PCA analysis for AKI and Control samples. **(B)** Volcano plot showing DEGs between AKI and Control samples. The *x*-axis represents the log2(FC), and the *y*-axis represents the -log10 (adjusted *p*-value). The blue dots represent downregulated genes, and the red dots represent upregulated genes. **(C)** The heatmap of gene network visualization and the branches of the dendrogram correspond to gene modules. **(D)** The correlation co-efficient and corresponding *p*-value between groups. **(E)** The heatmap of the relationship between each module. **(F)** The scatter plot of gene membership in turquoise module and gene significance in AKI. Most genes are clustered in the upper right corner, indicating that the genes in this module are highly correlated with AKI. **(G)** The scatter plot of gene membership in blue module and gene significance in AKI. **(H)** The Veen diagram of most significant WGCNA module (turquoise and blue) genes and DEGs. AKI, acute kidney injury; GSE, gene expression omnibus series; DEGs, differentially expressed genes; WGCNA, weighted gene co-expression network analysis; PCA, principal component analysis.

The workflow of the specific analysis is shown in Figure 1. Firstly, we reanalyzed our previously uploaded dataset GSE192883 (Supplementary Table S1). We combined IRI 28min and IRI 30min group into AKI group, and performed differential gene analysis on these 6 AKI samples and 3 Control samples. Figure 2A showed the principal component analysis (PCA) of these 9 samples, indicating a good distinction between AKI and the Control group. We got 2946 DEGs, including 1399 upregulated genes and 1547 downregulated genes (Supplementary Table S2). The volcano plot clearly presented the expression of all genes between each group, and the top 20 genes with lowest *p*-values were labeled (Figure 2B).

Then we performed WGCNA analysis on GSE192883 dataset to achieve key modules and genes. After determining the weighting coefficients, the disTOM of 5000 genes was obtained (Figure 2C), and 7 modules, each module represented by a different color. The heatmap displayed the relationship between module eigenvalues and AKI, each column showing the correlation coefficient and the corresponding *p*-value (Figure 2D). Red represented positive correlations, and blue represented negative correlations, and the darker the color, the larger the correlation coefficient. Figure 2E displayed the clusters and correlation of module eigengenes.

As we can see, turquoise and blue modules had the greatest correlation with AKI, and their correlation coefficients were 0.96 and 0.8, respectively. Therefore, we visualized the correlation between genes in these two modules and AKI **(** Figures 2F, G), and it could be seen that most genes of these two modules clustered in the upper right corner, indicating that the module attributes of these genes and their correlation with AKI were high. We took the genes of these two modules as the key genes obtained by WGCNA analysis, and the total number of genes was 4217 (Supplementary Table S3). The Venn diagram in Figure 2H illustrated the genes obtained through both WGCNA and DEGs analyses, revealing a total of 2123 overlapping genes.

### Screening of genes associated with AKI in GSE153625 dataset

**FIGURE 3:**
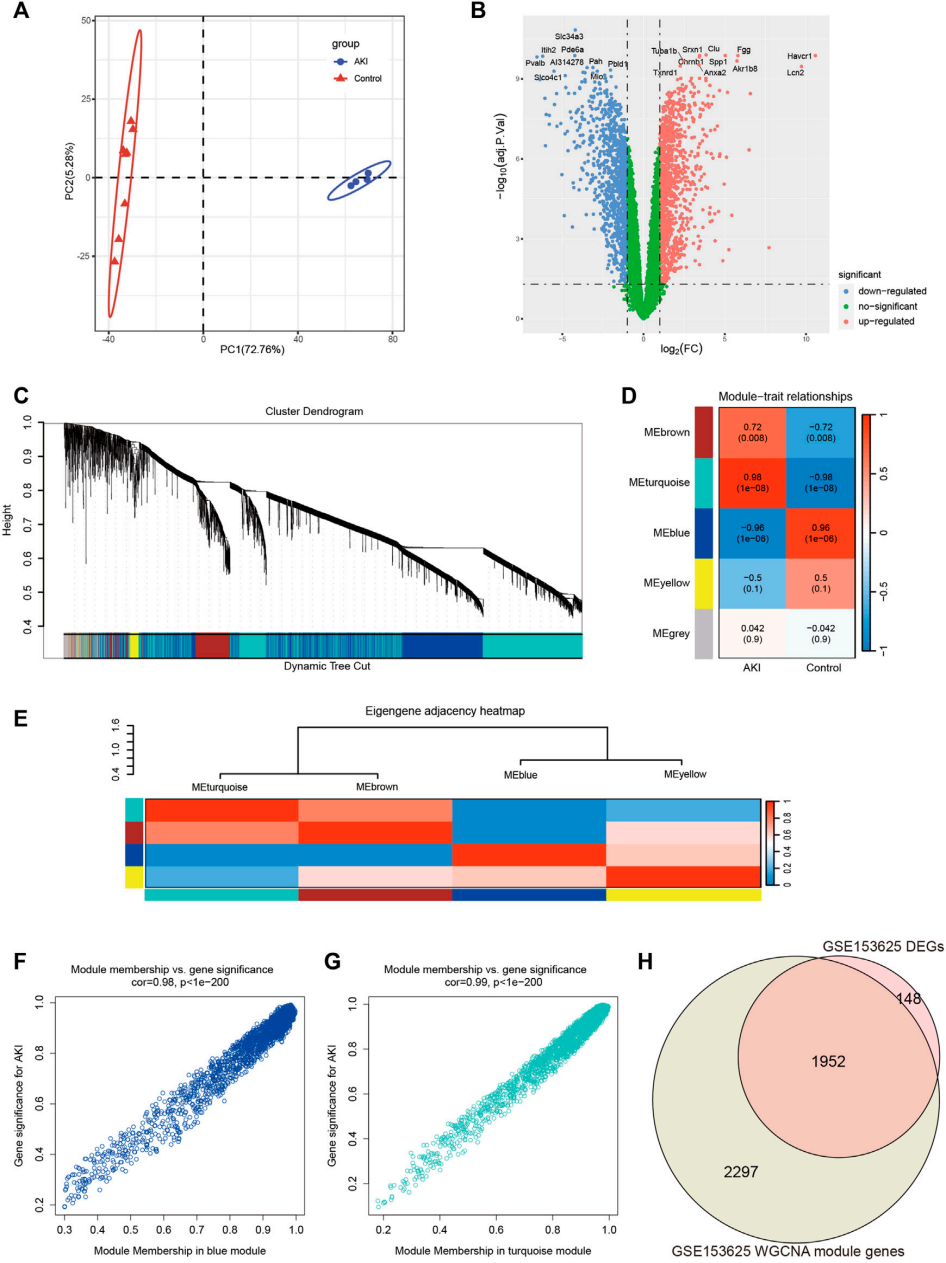
Screening of genes associated with AKI in GSE153625 dataset. **(A)** PCA analysis for AKI and Control samples. **(B)** Volcano plot showing DEGs between AKI and Control samples. **(C)** The branches of the dendrogram correspond to gene modules. **(D)** The correlation co-efficient and corresponding *p*-value between groups. **(E)** The heatmap of the relationship between each module. **(F)** The scatter plot of gene membership in blue module and gene significance in AKI. **(G)** The scatter plot of gene membership in turquoise module and gene significance in AKI. **(H)** The Veen diagram of most significant WGCNA module (turquoise and blue) genes and DEGs. AKI, acute kidney injury; GSE, gene expression omnibus series; DEGs, differentially expressed genes; WGCNA, weighted gene co-expression network analysis; PCA, principal component analysis.

In order to obtain more reliable and robust results, we downloaded another dataset GSE153625 (Supplementary Table S4). Figure 3A showed the PCA diagram of samples in GSE153625, indicating a good distinction between AKI and the Control group. We got 2100 DEGs (Supplementary Table S5), including 1035 upregulated genes and 1065 downregulated genes. The volcano plot clearly presented the expression of all genes between each group, and the top 20 genes with lowest *p*-values were labeled (Figure 3B).

Then we performed WGCNA analysis on GSE153625 dataset to achieve key modules and genes. After determining the weighting coefficients, the disTOM of 5000 genes was obtained (Figure 3C), and 5 modules, each module represented by a different color. The heatmap displayed the relationship between module eigenvalues and AKI, each column showing the correlation coefficient and the corresponding *p*-value (Figure 3D). Figure 3E displayed the clusters and correlation of module eigengenes. As we can see, turquoise and blue modules had the greatest correlation with AKI, and their correlation coefficients were 0.98 and 0.96, respectively. Therefore, we visualized the correlation between genes in these two modules and AKI (Figures 3F, G), and it could be seen that most genes in these two modules clustered in the upper right corner, indicating that the module attributes of these genes and their correlation with AKI were high. We took the genes of these two modules as the key genes obtained by WGCNA analysis, and the total number of genes was 4249 (Supplementary Table S6). Figure 3H showed the veen map of genes obtained by WGCNA analysis and DEGs analysis, and there were 1952 overlapping genes.

### Identification of common DEGs and hub genes in GSE192883 and GSE153625 datasets

**FIGURE 4:**
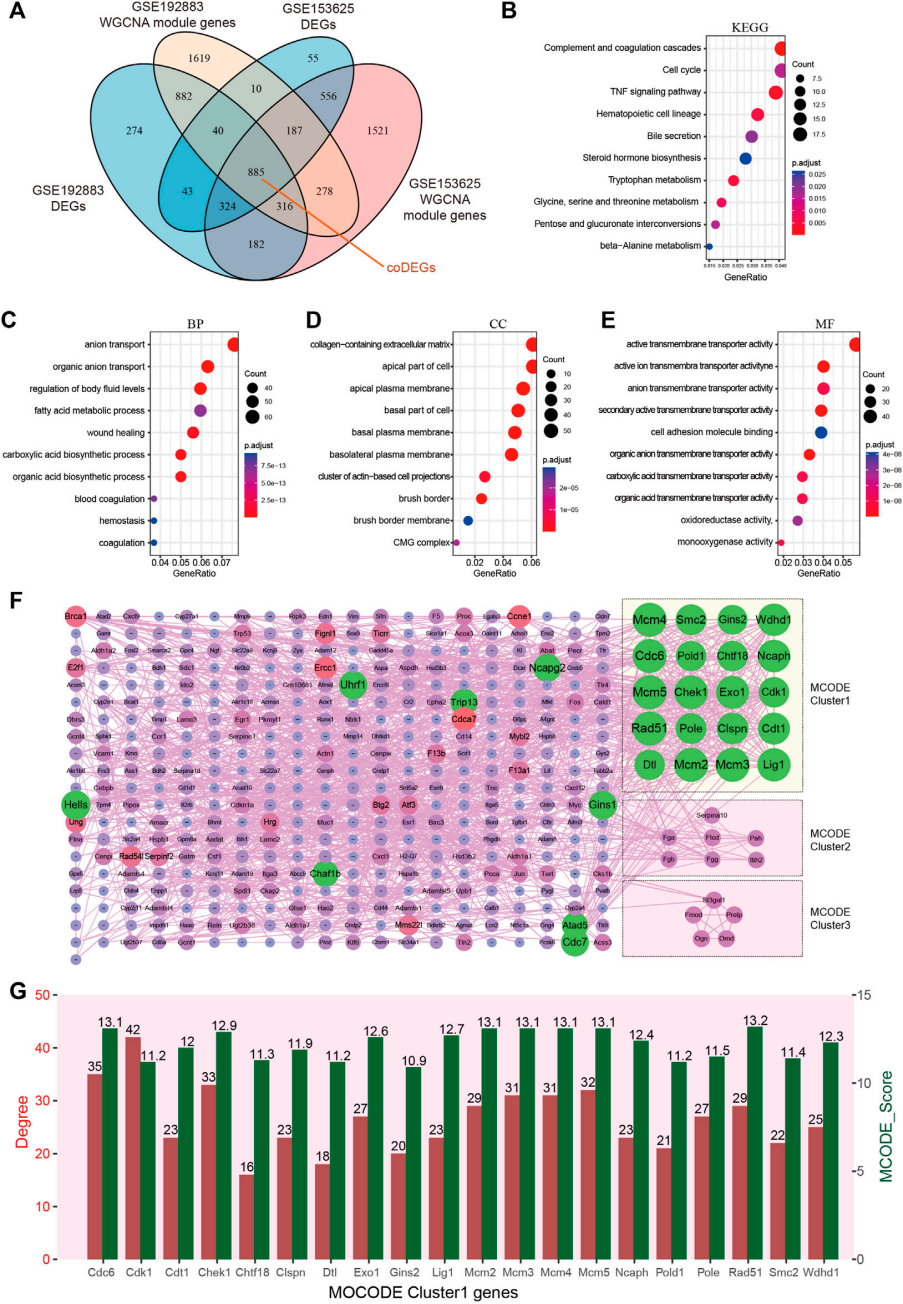
Identification of common DEGs and hub genes in GSE192883 and GSE153625 datasets. **(A)** The Veen diagram of DEGs and WGCNA module genes in GSE192883 and GSE153625 datasets. **(B)** KEGG pathway analysis of the 885 coDEGs. **(C–E)** GO analysis of the 885 coDEGs, including biological process (BP), cellular component (CC), and molecular function (MF) respectively. **(F)** PPI network reveals that coDEGs interact with each other, and the most significant three modules are visualized using MCODE plug-in. **(G)** The column shows the gene nodes degree and MCODE score of 20 hub genes of MCODE Cluster 1. GSE, gene expression omnibus series; DEGs, differentially expressed genes; WGCNA, weighted gene co-expression network analysis; PCA, principal component analysis; coDEGs, common DEGs; PPI, protein–protein interaction; GO, gene ontology; KEGG, Kyoto Encyclopedia of Genes and Genomes; MCODE, molecular complex detection.

Key genes were found in two datasets by limma and WGCNA analysis, as shown in Figure 4A, after Venn analysis, there were 885 genes expressed in all 4 gene clusters, named as common DEGs (coDEGs). Enrichment analysis were performed to reveal the role of the 885 coDEGs in AKI. KEGG pathway analysis (Figure 4B) showed that, these genes were mainly enriched in Complement and coagulation cascades, Cell cycle, and TNF signal pathway. GO analysis revealed that coDEGs in BP were mainly enriched in anion transport, and fatty acid metabolic process (Figure 4C); The CC were mainly enriched in collagen-containing extracellular matrix, and apical part of cell (Figure 4D); The MF were mainly enriched in active transmembrane transporter activity, and oxidoreductase activity (Figure 4E). The PPI network of coDEGs revealed that coDEGs interact with each other, and the most significant three modules were visualized using MCODE plug-in (Figure 4F). Figure 4G showed the gene nodes degree and MCODE score of 20 hub genes of MCODE Cluster 1. These 20 genes Cdc6, Cdk1, Cdt1, Chek1, Chtf18, Clspn, Dtl, Exo1, Gins2, Lig1, Mcm2, Mcm3, Mcm4, Mcm5, Ncaph, Pold1, Pole, Rad51, Smc2, and Wdhd1 in MOCDE 1 were hub genes.

### Immune cell infiltration analysis between AKI and control

**FIGURE 5:**
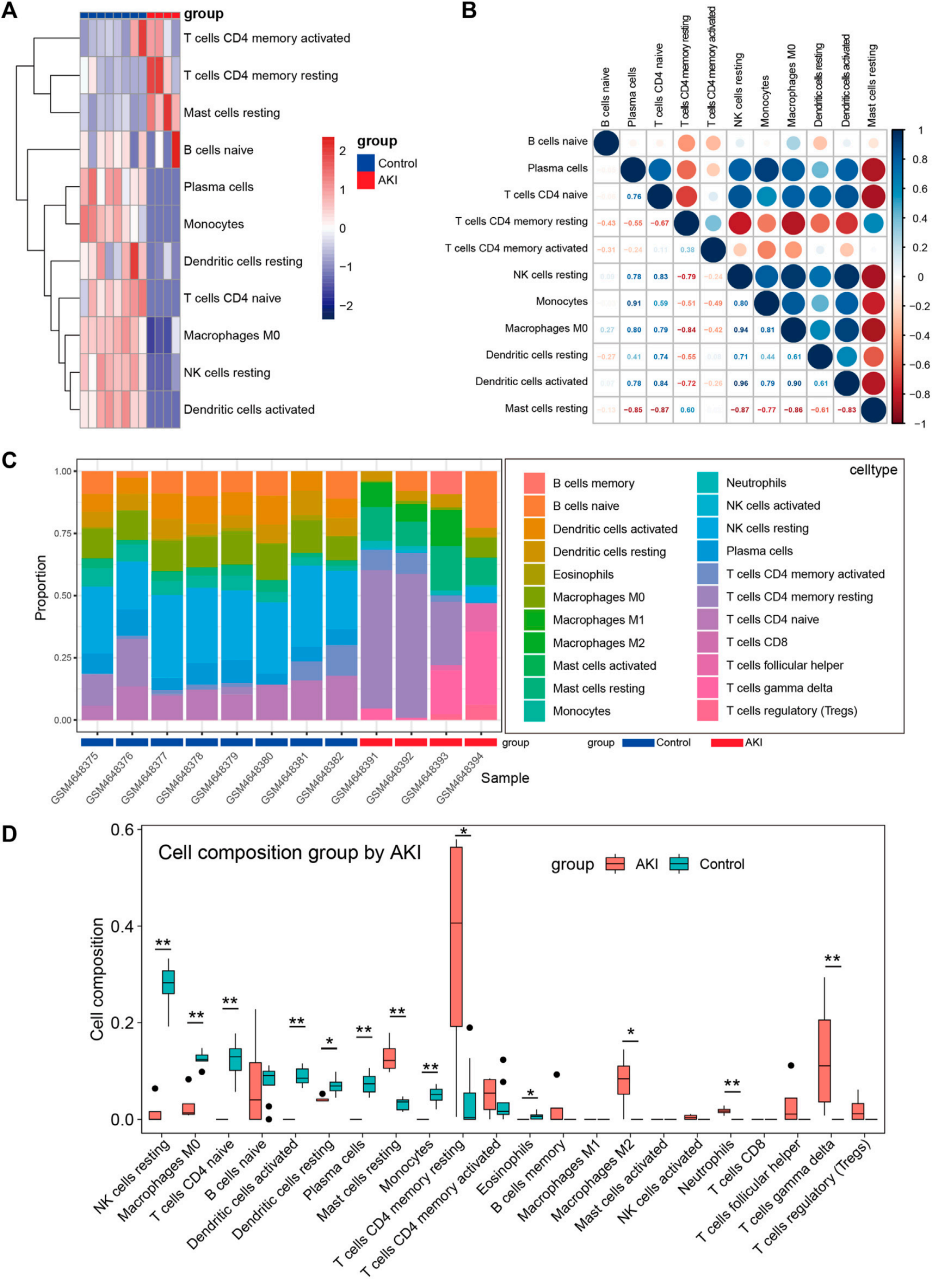
Immune cell infiltration analysis between AKI and Control group of GSE153625 dataset. **(A)** The heatmap of different immune cells expressed in each sample. **(B)** Correlation of 22 immune cell type compositions. **(C)** The proportion of 22 kinds of immune cells in different samples visualized from the barplot. **(D)** Comparison of different kinds of immune cells between AKI and Control groups. AKI, acute kidney injury; GSE, gene expression omnibus series.

Many reports have shown that immune changes are prominent during the occurrence and progression of AKI (Allison, 2018; do Valle Duraes et al., 2020; Melo Ferreira et al., 2021), so we paied special attention to the immune infiltration in AKI. We performed immune infiltration analysis on GSE153625 dataset, Figure 5A showed the heatmap of different immune cells expressed in each sample, Figure 5B presented the correlation of 22 kinds of immune cell type compositions. Figure 5C displayed the proportion of 22 kinds of immune cell type in each sample. Figure 5D illustrated the comparison of different kinds of immune cells between AKI and Control groups. These results demonstrated that AKI mice had a higher level of gamma delta T cells, CD4 memory resting cells T cells, resting mast cells, and M2 macrophages. The correlation of 22 types of immune cells revealed that CD4 memory resting cells T cells were positively associated with resting mast cells (r = 0.60), and that plasma cells were positively correlated with monocytes (r = 0.91). In general, various kinds of immune cells were differentially infiltrated in AKI, which could serve as the potential regulation point for AKI treatment.

### Identification of deFRGs in AKI

**FIGURE 6:**
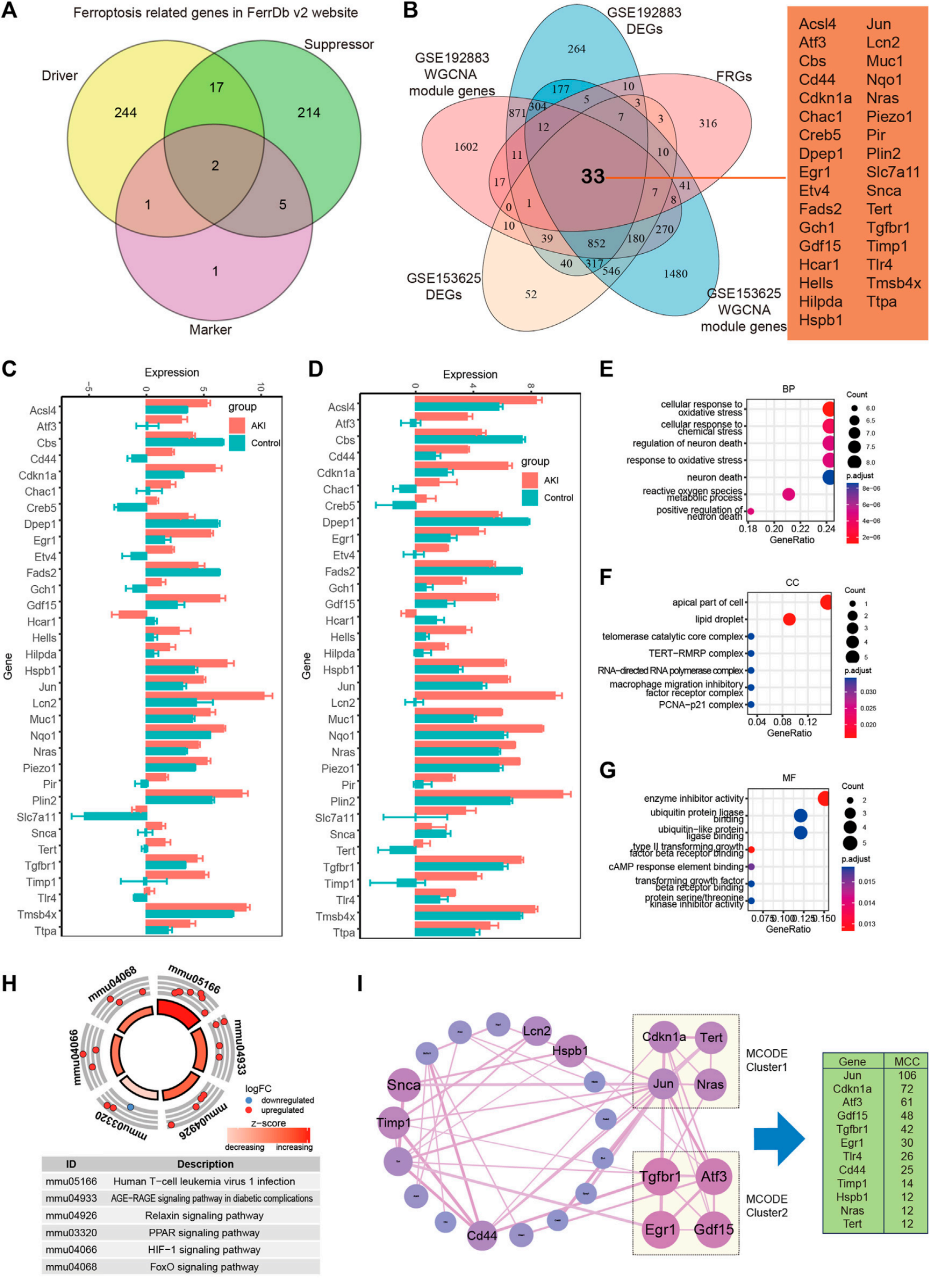
Identification of differentially expressed FRGs in GSE192883 and GSE153625 datasets. **(A)** The Veen diagram of Ferroptosis-related genes (FRGs) in FerrDb v2 website. **(B)** The Veen diagram of 33 differentially expressed FRGs (deFRGs) in GSE192883 and GSE153625 datasets. **(C)** The expression of 33 deFRGs in GSE192883 dataset. **(D)** The expression of 33 deFRGs in GSE153625 dataset. **(E–G)** GO analysis of the 33 deFRGs, including biological process (BP), cellular component (CC), and molecular function (MF) respectively. **(H)** KEGG pathway analysis of the 33 deFRGs. **(I)** PPI network reveals that deFRGs interact with each other, and the most significant two modules are visualized by using MCODE plug-in. The column shows the MCC score of top 12 deFRGs. FRGs, ferroptosis-related genes; deFRGs, differentially expressed FRGs; PPI, protein–protein interaction; GO, gene ontology; KEGG, Kyoto Encyclopedia of Genes and Genomes; MCODE, molecular complex detection.

In order to recognize ferroptosis-related genes in AKI, 484 unique FRGs were selected from FerrDb V2 database (Figure 6A), and they were overlapped with the DEGs and turquoise of GSE192883 and GSE153625 datasets. The results showed that there were 33 deFRGs commonly differentially expressed in both datasets (Figure 6B). Fads2, Dpep1, Cbs, and Hcar1 were downregulated in both datasets, Snca were upregulated in GSE192883 while downregulated in GSE153625, and other deFRGs were upregulated in both datasets (Figures 6C,D). The role of these 33 deFRGs was explored by functional enrichment analysis. The top 7 GO items under each classification were shown in bar charts (Figures 6E–G). The results revealed that these deFRGs were involved in lipid droplet, cellular response to oxidative stress, and enzyme inhibitor activity. The KEGG pathway enrichment analysis indicated significant enrichment of deFRGs in the terms of Human T cell leukemia virus 1 infection, AGE−RAGE signaling pathway in diabetic complications, and HIF1 signaling pathway (Figure 6H). Furthermore, PPI analysis demonstrated interactions among deFRGs. Cdkn1a, Tert, Jun and Nras were grouped into MCODE cluster 1 with Jun having the highest MCC score (Figure 6I).

### Screen for diagnostic markers in two human datasets by machine learning

**FIGURE 7:**
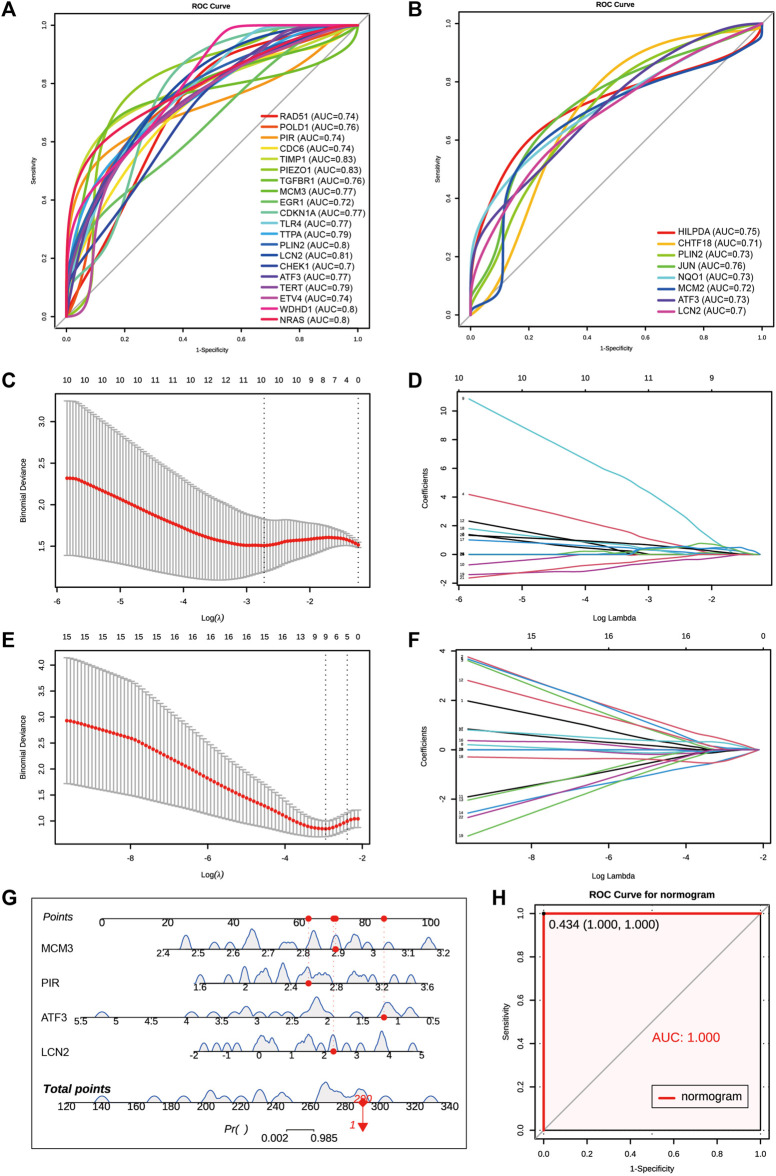
Screen for diagnostic markers in two human datasets by machine Learning. **(A)** The ROC plot of 20 upregulated hub genes with AUC over than 0.7 in GSE217427 human dataset. **(B)** The ROC plot of 8 upregulated hub genes with AUC over than 0.7 in GSE139061 human dataset. **(C,D)** Lasso analysis of the 25 hub genes with AUC over than 0.7 in GSE217427 human dataset. **(E,F)** Lasso analysis of the 25 hub genes with AUC over than 0.7 in GS E139061 human dataset. **(G)** The diagnostic nomogram model of final 4 hub genes ATF3,LCN2, MCM3, and PIR in GSE139061 human dataset. The red dot and line represent one of the AKI samples. **(H)** The ROC curve of the diagnostic nomogram model GSE139061 human dataset, the AUC of this model is 1. AKI, acute kidney injury; GSE, gene expression omnibus series; ROC, receiver operating characteristic; AUC, area under the curve; Lasso, least absolute shrinkage and selection operator.

We combined 20 MCODE cluster 1 genes of coDEGs and 33 deFRGs as hub genes for AKI. Among these 53 hub genes, there were 48 genes upregulated in both GSE192883 and GSE153625 datasets (Table 3). We then performed ROC analysis on these 48 upregulated hub genes in two human datasets GSE217427 and GSE139061. The results showed that there were 20 hub genes with AUC over than 0.7 in GSE217427 human dataset (Figure 7A), while there were 8 hub genes with AUC over than 0.7 in GSE139061 human dataset (Figure 7B). Among these 25 genes with AUC over than 0.7, LCN2, PLin2, and ATF3 had an AUC over than 0.7 in both two human datasets. Further machine learning analysis (Lasso analysis) was performed on these 25 hub genes in the GSE217427 (Figures 7C, D) and GSE139061 human datasets (Figures 7E, F). Through Lasso analysis, ATF3, CHEK1, ETV4, LCN2, MCM3, NRAS, PIEZO1, PIR, TERT, and WDHD1 were identified in GSE217427. In GSE139061 dataset, ATF3, CHTF18, EGR1, HILPDA, LCN2, MCM3, NQO1, PIR, TIMP1 were screened. Four hub genes (LCN2, ATF3, PIR, and MCM3) were found in both datasets. A diagnostic nomogram model was constructed using these final four hub genes from the GSE139061 human dataset to predict an individual’s risk of developing AKI. In addition, the ROC curve of the diagnostic nomogram model was depicted in the GSE139061 human dataset to assess its diagnostic ability (Figure 7G). The AUC of this model was 1 (Figure 7H), indicating that this diagnostic nomogram model of LCN2, ATF3, PIR, and MCM3, can effectively distinguish AKI samples. Therefore, we hypothesize that these genes are highly potential biomarkers for AKI.

**TABLE 3:** The list of 48 hub genes upregulated in both GSE192883 and GSE153625 datasets.

Gene name	GSE192883			GSE153625			Source of hub genes
	logFC	adj.P.Val	WGCNA	logFC	adj.P.Val	WGCNA	
Lcn2	5.888	0.000	turquoise	9.722	0.000	turquoise	deFGRs
Timp1	5.257	0.000	turquoise	5.435	0.000	turquoise	deFGRs
Slc7a11	4.500	0.000	turquoise	3.449	0.009	blue	deFGRs
Egr1	4.012	0.000	turquoise	1.967	0.000	blue	deFGRs
Gdf15	3.709	0.000	turquoise	3.383	0.000	turquoise	deFGRs
Etv4	3.516	0.000	turquoise	2.262	0.000	turquoise	deFGRs
Cd44	3.419	0.000	turquoise	2.212	0.000	turquoise	deFGRs
Creb5	3.300	0.000	turquoise	2.233	0.005	turquoise	deFGRs
Cdc6	3.178	0.004	blue	2.647	0.001	turquoise	coDEGs in Cluster 1
Atf3	2.933	0.001	turquoise	3.889	0.000	turquoise	deFGRs
Hspb1	2.839	0.000	turquoise	3.174	0.000	turquoise	deFGRs
Cdkn1a	2.836	0.000	turquoise	4.192	0.000	turquoise	deFGRs
Exo1	2.732	0.019	blue	2.397	0.002	turquoise	coDEGs in Cluster 1
Dtl	2.686	0.018	blue	2.755	0.000	blue	coDEGs in Cluster 1
Clspn	2.607	0.010	blue	2.310	0.004	turquoise	coDEGs in Cluster 1
Plin2	2.604	0.000	turquoise	3.661	0.000	turquoise	deFGRs
Gch1	2.404	0.000	turquoise	2.499	0.000	turquoise	deFGRs
Hells	2.250	0.010	blue	2.787	0.000	turquoise	deFGRs
Chek1	2.186	0.012	blue	2.704	0.000	turquoise	coDEGs in Cluster 1
Cdk1	2.169	0.005	blue	1.353	0.000	blue	coDEGs in Cluster 1
Rad51	2.124	0.027	blue	1.243	0.001	turquoise	coDEGs in Cluster 1
Pir	2.104	0.000	turquoise	2.010	0.000	turquoise	deFGRs
Mcm3	2.087	0.010	blue	1.990	0.000	turquoise	coDEGs in Cluster 1
Chtf18	1.987	0.012	blue	1.305	0.007	turquoise	coDEGs in Cluster 1
Ncaph	1.951	0.008	blue	2.007	0.007	turquoise	coDEGs in Cluster 1
Gins2	1.877	0.006	blue	1.963	0.000	turquoise	coDEGs in Cluster 1
Ttpa	1.870	0.002	turquoise	1.052	0.002	turquoise	deFGRs
Mcm5	1.859	0.018	blue	1.967	0.000	turquoise	coDEGs in Cluster 1
Lig1	1.799	0.006	blue	1.352	0.000	turquoise	coDEGs in Cluster 1
Chac1	1.795	0.012	turquoise	2.665	0.000	turquoise	deFGRs
Jun	1.791	0.000	turquoise	1.771	0.000	blue	deFGRs
Mcm4	1.775	0.004	blue	1.475	0.000	turquoise	coDEGs in Cluster 1
Acsl4	1.762	0.000	turquoise	2.587	0.000	blue	deFGRs
Tert	1.761	0.001	turquoise	2.200	0.001	blue	deFGRs
Cdt1	1.675	0.006	blue	1.204	0.000	turquoise	coDEGs in Cluster 1
Pole	1.649	0.035	blue	1.321	0.002	turquoise	coDEGs in Cluster 1
Wdhd1	1.624	0.003	blue	1.995	0.000	turquoise	coDEGs in Cluster 1
Pold1	1.605	0.000	blue	1.163	0.000	turquoise	coDEGs in Cluster 1
Mcm2	1.544	0.008	blue	1.191	0.000	turquoise	coDEGs in Cluster 1
Muc1	1.541	0.002	turquoise	1.951	0.000	blue	deFGRs
Hilpda	1.367	0.009	turquoise	1.481	0.001	turquoise	deFGRs
Smc2	1.326	0.016	blue	1.228	0.000	turquoise	coDEGs in Cluster 1
Tlr4	1.285	0.002	turquoise	1.042	0.004	blue	deFGRs
Tmsb4x	1.219	0.002	turquoise	1.018	0.000	turquoise	deFGRs
Nqo1	1.148	0.000	turquoise	2.659	0.000	turquoise	deFGRs
Nras	1.126	0.000	turquoise	1.133	0.000	blue	deFGRs
Piezo1	1.074	0.002	turquoise	1.411	0.000	blue	deFGRs
Tgfbr1	1.016	0.020	turquoise	1.265	0.000	blue	deFGRs

GSE, gene expression omnibus series; WGCNA, weighted gene co-expression network analysis; coDEGs, common differentially expressed genes; deFRGs, differentially expressed ferroptosis-related genes; FC, fold change.

### Validation of 4 key diagnostic signature genes

**FIGURE 8:**
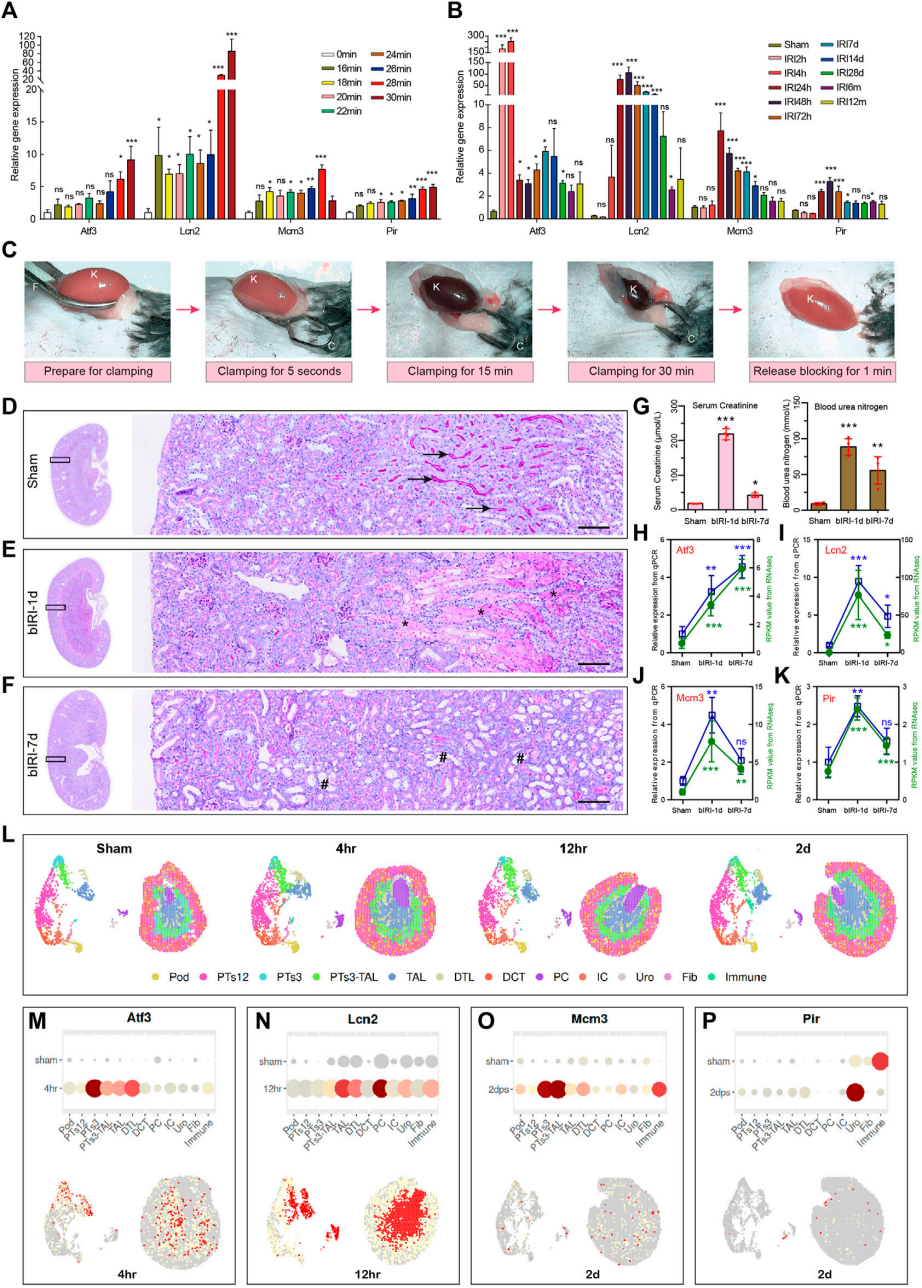
Validation of expression patterns of 4 key diagnostic signature genes. **(A)** The expression of 4 key diagnostic signature genes in different ischemia time of AKI in our previously uploaded dataset GSE192883. One-way ANOVA and Dunnett-t test, ns indicates no significant, *p < 0.05, **p < 0.01, ***p < 0.001, compared to 0min group. **(B)** The expression of 4 key diagnostic signature genes in different reperfusion time of AKI in dataset GSE98622. One-way ANOVA and Dunnett-t test, ns indicates no significant, *p < 0.05, **p < 0.01, ***p < 0.001, compared to Sham group. **(C)** The procedure of the mouse renal pedicle clamping before, during, and after renal ischemia reperfusion surgery. K for kidney, F for curved forcep, C for microvascular clamp. **(D–F)** Representative micrographs of PAS staining shows the pathological features of kidney injury at different reperfusion times: Sham group **(D)**, bIRI-1d group **(E)**, bIRI-7d group **(F)**. Scale bars: 100 μm. The arrow shows the brush border of normal tubule, the asterisk (*) shows the blocked tubule, and the well sign (#) represents a dilated renal tubule that has completely shed its epithelial cells. **(G)** The serum creatinine (left) and the blood urine nitrogen (right) levels in different groups. *p < 0.05, **p < 0.01, ***p < 0.001, compared to Sham group. **(H–K)** The expression level of Atf3 **(H)**, Lcn2 **(I)**, Mcm3 **(J)**, and Pir **(K)**. The expression level of all four genes are highly consistent between RNA-seq data and qPCR results. One-way ANOVA and Dunnett-t test, ns indicates no significant, *p < 0.05, **p < 0.01, ***p < 0.001, compared to Sham group. **(L)** Single cell sequencing and spatial transcriptomics analysis of acute kidney injury in mouse (GSE182939). Podocytes (Pod), proximal tubule segments 1-3 (PTS1-3), descending and ascending thin limp of loop of Henle (DTL, TAL), distal convoluted tubule (DCT), principal cells (PC1-2), intercalated cells (IC), urothelium (Uro), fibroblasts (Fib), Immune cells (Immune). **(M–P)** Single cell sequencing and spatial transcriptomics showing the expression of the four key genes in mouse IRI. The highest gene expression time point is selected to present in the figure. AKI, acute kidney injury; GSE, gene expression omnibus series; bIRI, bilateral ischemia reperfusion injury; qPCR, quantitative real-time polymerase chain reaction.

To validate the above results and expression patterns of these four key diagnostic signature genes, we first reanalyzed our previously uploaded dataset GSE192883 and another AKI dataset GSE98622 to demonstrate the mRNA expression of these four genes at different ischemia times and different reperfusion times. We found that these four genes began to upregulate within 16–18 min of ischemia, indicating their sensitivity to mild ischemia. The expression level of severe ischemia was higher in Ischemia 28 min, indicating a positive correlation with the degree of ischemia (Figure 8A). The results of IRI-AKI with different reperfusion (Figure 8B) showed that the Atf3 gene began to express rapidly at 2 h after ischemia, which could predict the occurrence of AKI early and its expression remained elevated even after 24 h. The expression of Lcn2 gradually increased at 4 h after reperfusion, peaked at 24–72 h, and remained elevated at 7d after reperfusion. The expressions of Pir and Mcm3 began to increase at 24 h and reached a peak at 24–48 h. These results indicated that all four genes were highly responsive to AKI with varying degrees of ischemia and time of reperfusion. Secondly, we conducted our own bilateral IRI (bIRI) model, as shown in Figures 8C–G, the pathological injury was severe 1 day after bIRI and persisted for 7 days. The qPCR results (Figures 8H–K) demonstrated a high level of consistency between RNA-seq data and qPCR results. Finally, we utilized the Kidney Interactive Transcriptomics (KIT) online tools (http://humphreyslab.com/SingleCell/) to identify the expression pattern of these four genes through single cell sequencing and spatial transcriptomic analysis in GSE182939 (Dixon et al., 2022) (Figure 8L). The results (Figure 8M-P) showed that, Atf3 was mainly expressed in proximal tubule segments 3 to descending thin limp of loop of Henle, with the highest expression at 4 h after IRI; Lcn2 was mainly expressed in the descending and ascending thin limp of loop of Henle, as well as principal cells, with the highest expression at 12 h after IRI; Mcm3 was mainly expressed in proximal tubule segments 3 to the descending thin limp of loop of Henle, with the highest expression at 2d after IRI; Pir was mainly expressed in urothelium, with the highest expression at 2d after IRI.

## Discussion

Although AKI is associated with high morbidity and mortality, therapeutic options for AKI are still limited, and the underlying mechanisms of AKI remain largely unclear. Traditional diagnostic methods such as serum creatinine and urine output may not be sufficient for early diagnosis (Zhang et al., 2022). Therefore, identifying new biomarkers before kidney dysfunction occurs could help detect AKI earlier and play a critical role in developing new therapies for its treatment (Chen et al., 2022). Ferroptosis is a form of iron-dependent regulated necrosis characterized by intracellular iron retention, depletion of reduced GSH, and accumulation of lipid ROS (Martin-Sanchez et al., 2017). It mainly affects three metabolic pathways: iron metabolism, lipid metabolism, and amino acid metabolism. Recently, ferroptosis has been reported to be involved in AKI (Martin-Sanchez et al., 2017; Feng et al., 2022).

In the present study, we aimed to identify novel diagnostic FRGs genes for AKI based on bioinformatics and machine learning. We analyzed our previously uploaded dataset GSE192883 (Chen et al., 2022) and another mouse dataset GSE153625 (Kim et al., 2020) from GEO database using limma and WGCNA analysis, and identified 885 coDEGs. Enrichment analysis showed that these genes were mainly enriched in Complement and Coagulation Cascades, Cell Cycle, and Oxidoreductase Activity. Recent studies have shown that the complement system is activated in pediatric patients with AKI, and complement proteins may serve as biomarkers and therapeutic targets for AKI (Stenson et al., 2023). Our recently study has reported that EGR1 increased SOX9 expression in renal tubular epithelial cells by directly binding to the promoter of the Sox9 gene, thereby promoting proliferation of SOX9^+^ cells after AKI (Chen et al., 2022). This indicated that pathways mentioned above were crucial in the occurrence and development of AKI. Additionally, we performed a PPI network analysis on 885 coDEGs and identified 20 hub genes using MCODE.

We also performed immune infiltration analysis to identify the immune changes in AKI. The results showed that AKI mice had a higher levels of gamma delta T cells (Hochegger et al., 2007), CD4 memory resting cells T cells (Farooqui et al., 2023), resting mast cells (van der Elst et al., 2023), and M2 macrophages (Li et al., 2018; Singbartl et al., 2019). It has been reported that the gamma delta T cells played a role as mediator cells in the first 72 h of renal IRI (Hochegger et al., 2007). Observed an increase in CD4 memory T cells in patients who developed immune checkpoint inhibitor-related AKI (Farooqui et al., 2023). Mast cells had potential protective effects on tissue remodeling post-injury (van der Elst et al., 2023). These immune cells were differentially infiltrated in AKI, which could serve as a potential regulatory point for AKI treatment.

In order to recognize differentially expressed ferroptosis-related genes in AKI, 484 unique FRGs were overlapped with the DEGs and turquoise of GSE192883 and GSE153625 datasets. As a result, 33 deFRGs were commonly differentially expressed in both datasets. These deFRGs are involved in lipid droplet formation, cellular response to oxidative stress, and Human T cell leukemia virus 1 infection. Numerous studies have reported that lipid droplet formation and cellular response to oxidative stress are associated with ferroptosis (Bai et al., 2019; Zou et al., 2019; Xin et al., 2022). For examples, in renal cancer, MS4A15 regulates anti-ferroptotic lipid reservoirs to provide a key resistance mechanism that is distinct from antioxidant and lipid detoxification pathways (Xin et al., 2022). HIF-2α selectively enriches polyunsaturated lipids, which are the rate-limiting substrates for lipid peroxidation, by activating the expression of hypoxia-inducible, lipid droplet-associated protein (Zou et al., 2019). Further PPI analysis found 20 hub genes including Cdkn1a, Tert, Jun, Nras, and Jun with the biggest MCC score.

Then, we analyzed the diagnostic value of 48 upregulated hub genes in AKI based on ROC analysis and machine learning. Among them, 25 genes had an AUC greater than 0.7, and LCN2, PLIN2, and ATF3 had an AUC greater than 0.7 in both two human datasets. Using Lasso analysis, four hub genes (LCN2, ATF3, PIR, and MCM3) were identified in both human datasets. Through validation in the human dataset GSE217427, a diagnostic nomogram model constructed using these final four hub genes was able to effectively distinguish AKI samples. Finally, the expression of LCN2, ATF3, PIR, and MCM3 was validated in AKI datasets and laboratory investigations. These four genes may serve as ideal markers for ferroptosis in AKI. Our findings provide novel insights into critical genes involved in the progression of AKI, which can guide individualized diagnosis and treatment.

Lcn2 also named as neutrophil gelatinase associated lipoprotein (NGAL), was reported closely associated with AKI by several experimental and clinical studies (Cowland and Borregaard, 1997; Schmidt-Ott et al., 2006). Lcn2 was expressed at low levels in kidney under normal conditions, while increased significantly within 2–6 h after AKI (Koyner et al., 2010; Delcroix et al., 2013). Lcn2 level was closely associated with the severity of kidney injury, and more accurate for predicting AKI development than creatinine (Jahaj et al., 2021). Chui et al. demonstrated that the AUC of Lcn2 was over 0.73 to detect AKI at 3 days before AKI onset (Chui et al., 2020). In our study, the AUCs of Lcn2 were 0.81 and 0.7 in human datasets GSE217427 and GSE139061.

Atf3, the full name is activation transcription factor 3, is a member of the ATF/CREB subfamily of the basic-region leucine zipper family. Atf3 signaling pathway acted as protective role in attenuating inflammation and ischemia reperfusion induced tubular cell death and nephrotoxicity (Cheng and Lin, 2011). Atf3 was reported plays an important role in cell ferroptosis, knockdown of Atf3 could significantly increase the levels of SLC7A11, GPX4 and increased the cell viability (Wang et al., 2021). Integration of spatial and single-cell transcriptomics analysis found that Atf3 was acted as a chemotactic factor in S3 injured proximal tubular cells, which may be responsible for neutrophil chemotaxis (Melo Ferreira et al., 2021). In conclusion, Atf3 plays an important role in renal protection, and may serve as a potential novel diagnostic and therapeutic molecules in AKI.

Pir, the full name is Pirin, is a nonheme iron (Fe) binding nuclear protein, plays an important role in mediating ferroptosis resistance in human pancreatic cancer cells (Hu et al., 2021). Pir has been shown to modulate the binding affinity between p65 homodimeric NF-κB and κB DNA. Binding of the Fe(III) form of Pirin to the p65-DNA complex significantly alters both the conformational dynamics of the DNA and the interactions between p65 and the DNA (Barman and Hamelberg, 2016). Orzaez et al. (Orzaez et al., 2001) reported that Pir can stabilize the formation of quaternary complexes between Bcl-3, the anti-apoptotic transcription factor NF-κB and its DNA target sequences *in vitro*. Licciulli reported that Pir was required for terminal myeloid maturation, and its downregulation may contribute to the differentiation arrest associated with acute myeloid leukemia (Licciulli et al., 2010). However, its role in kidney has not yet been reported. Thus, the regulatory role of Pir in AKI needs to be further investigated in functional and mechanistic studies.

Minichromosome maintenance (MCM) proteins are DNA-dependent ATPases that bind to replication origins and restrict DNA synthesis to a single round of DNA replication. They can reflect the cell cycle status due to their stable state during the cell cycle and proteolysis in quiescent cells (G0). One member of this family, Mcm3, is reportedly active in most cancers (Ha et al., 2004; Söling et al., 2005). Mcm3 was reported regulates the assembly and activity of MCM2-7 complex by phosphorylated at Ser-112 by Cdk1, can directly combine with cyclin D1 and participate in the regulation of cell proliferation, and had a strong pro-apoptotic effect (Ji et al., 2017). However, the role of Mcm3 in kidney has not yet been reported. It is important to note that further clarification is required for these four genes regarding their involvement in ferroptosis and AKI.

This research also has some limitations. Firstly, the research was mainly conducted based on online public databases; more external human data is needed to verify our model. Secondly, we mainly focused on ferroptosis-related genes, and there may be more precise genes that were underestimated. Finally, it is necessary to establish cell models and animal models to further study the mechanism of these hub genes in AKI.

## Conclusion

In summary, our study systematically discovered three candidate hub genes associated with ferroptosis (Lcn2, Atf3, and Pir) and one coDEG (Mcm3). We also provided a nomogram for diagnosing AKI through various bioinformatics analyses and machine learning algorithms. The versatility of the signature was proven through internal verification, demonstrating its suitability for clinical use. Additionally, we identified dysregulated immune cell proportions in AKI. Our study has provided valuable information on potential ferroptosis-related genes as diagnostic candidates for AKI patients.

## Data Availability

The original contributions presented in the study are included in the article/Supplementary Material, further inquiries can be directed to the corresponding authors.

## References

[B1] Allison S. J. (2018). Immune networks in CI-AKI. Nat. Rev. Nephrol. 14 (9), 536. 10.1038/s41581-018-0036-0 29921980

[B2] Bader G. D. Hogue C. W. (2003). An automated method for finding molecular complexes in large protein interaction networks. BMC Bioinforma. 4, 2. 10.1186/1471-2105-4-2 PMC149346 12525261

[B3] Bai Y. Meng L. Han L. Jia Y. Zhao Y. Gao H. (2019). Lipid storage and lipophagy regulates ferroptosis. Biochem. BIOPHYSICAL Res. Commun. 508 (4), 997–1003. 10.1016/j.bbrc.2018.12.039 30545638

[B4] Barman A. Hamelberg D. (2016). Fe(II)/Fe(III) redox process can significantly modulate the conformational dynamics and electrostatics of Pirin in NF-κB regulation. ACS Omega 1 (5), 837–842. 10.1021/acsomega.6b00231 31457166 PMC6640773

[B5] Bellomo R. Kellum J. A. Ronco C. (2012). Acute kidney injury. Lancet 380 (9843), 756–766. 10.1016/s0140-6736(11)61454-2 22617274

[B6] Chen J. Chen Y. Olivero A. Chen X. (2020). Identification and validation of potential biomarkers and their functions in acute kidney injury. Front. Genet. 11, 411. 10.3389/fgene.2020.00411 32528518 PMC7247857

[B7] Chen J. W. Huang M. J. Chen X. N. Wu L. L. Li Q. G. Hong Q. (2022a). Transient upregulation of EGR1 signaling enhances kidney repair by activating SOX9(+) renal tubular cells. Theranostics 12 (12), 5434–5450. 10.7150/thno.73426 35910788 PMC9330523

[B8] Chen Y. L. Li H. K. Wang L. Chen J. W. Ma X. (2022b). No safe renal warm ischemia time-The molecular network characteristics and pathological features of mild to severe ischemia reperfusion kidney injury. Front. Mol. Biosci. 9, 1006917. 10.3389/fmolb.2022.1006917 36465563 PMC9709142

[B9] Cheng C. F. Lin H. (2011). Acute kidney injury and the potential for ATF3-regulated epigenetic therapy. Toxicol. Mech. Methods 21 (4), 362–366. 10.3109/15376516.2011.557876 21495874

[B10] Chin C. H. Chen S. H. Wu H. H. Ho C. W. Ko M. T. Lin C. Y. (2014). cytoHubba: identifying hub objects and sub-networks from complex interactome. BMC Syst. Biol. 8 (4), S11. 10.1186/1752-0509-8-s4-s11 25521941 PMC4290687

[B11] Chui H. Caldwell J. Yordanova M. Cockovski V. Fredric D. Harel-Sterling M. (2020). Tubular injury and cell-cycle arrest biomarkers to predict acute kidney injury in noncritically ill children receiving aminoglycosides. Biomarkers Med. 14 (10), 879–894. 10.2217/bmm-2019-0419 PMC8274558 32808826

[B12] Cowland J. B. Borregaard N. (1997). Molecular characterization and pattern of tissue expression of the gene for neutrophil gelatinase-associated lipocalin from humans. GENOMICS 45 (1), 17–23. 10.1006/geno.1997.4896 9339356

[B13] Delcroix G. Gillain N. Moonen M. Radermacher L. Damas F. Minon J. M. (2013). NGAL usefulness in the intensive care unit three hours after cardiac surgery. ISRN Nephrol. 2013, 865164. 10.5402/2013/865164 24967231 PMC4045423

[B14] Dixon E. E. Wu H. Muto Y. Wilson P. C. Humphreys B. D. (2022). Spatially resolved transcriptomic analysis of acute kidney injury in a female murine model. J. Am. Soc. Nephrol. 33 (2), 279–289. 10.1681/asn.2021081150 34853151 PMC8819997

[B15] do Valle Duraes F. Lafont A. Beibel M. Martin K. Darribat K. Cuttat R. (2020). Immune cell landscaping reveals a protective role for regulatory T cells during kidney injury and fibrosis. JCI Insight 5 (3), 130651. 10.1172/jci.insight.130651 32051345 PMC7098794

[B16] Fan J. Shi S. Qiu Y. Liu M. Shu Q. (2022). Analysis of signature genes and association with immune cells infiltration in pediatric septic shock. Front. Immunol. 13, 1056750. 10.3389/fimmu.2022.1056750 36439140 PMC9686439

[B17] Farooqui N. Zaidi M. Vaughan L. McKee T. D. Ahsan E. Pavelko K. D. (2023). Cytokines and immune cell phenotype in acute kidney injury associated with immune checkpoint inhibitors. Kidney Int. Rep. 8 (3), 628–641. 10.1016/j.ekir.2022.11.020 36938084 PMC10014345

[B18] Feng Q. Yu X. Qiao Y. Pan S. Wang R. Zheng B. (2022). Ferroptosis and acute kidney injury (AKI): Molecular mechanisms and therapeutic potentials. Front. Pharmacol. 13, 858676. 10.3389/fphar.2022.858676 35517803 PMC9061968

[B19] Friedman J. Hastie T. Tibshirani R. (2010). Regularization paths for generalized linear models via coordinate descent. J. Stat. Softw. 33 (1), 1–22. 10.18637/jss.v033.i01 20808728 PMC2929880

[B20] Ginestet C. (2011). ggplot2: Elegant graphics for data analysis. J. R. Stat. Soc. Ser. A-STATISTICS Soc. 174, 245–246. 10.1111/j.1467-985X.2010.00676_9.x

[B21] Ha S. A. Shin S. M. Namkoong H. Lee H. Cho G. W. Hur S. Y. (2004). Cancer-associated expression of minichromosome maintenance 3 gene in several human cancers and its involvement in tumorigenesis. Clin. CANCER Res. 10 (24), 8386–8395. 10.1158/1078-0432.Ccr-04-1029 15623617

[B22] Hochegger K. Schätz T. Eller P. Tagwerker A. Heininger D. Mayer G. (2007). Role of alpha/beta and gamma/delta T cells in renal ischemia-reperfusion injury. Am. J. Physiol. Ren. Physiol. 293 (3), F741–F747. 10.1152/ajprenal.00486.2006 17567936

[B23] Hosohata K. Harnsirikarn T. Chokesuwattanaskul S. (2022). Ferroptosis: A potential therapeutic target in acute kidney injury. Int. J. Mol. Sci. 23 (12), 6583. 10.3390/ijms23126583 35743026 PMC9223765

[B24] Hu N. Bai L. Dai E. Han L. Kang R. Li H. (2021). Pirin is a nuclear redox-sensitive modulator of autophagy-dependent ferroptosis. Biochem. BIOPHYSICAL Res. Commun. 536, 100–106. 10.1016/j.bbrc.2020.12.066 33373853

[B25] Huang M. Li D. Chen J. Ji Y. Su T. Chen Y. (2022). Comparison of the treatment efficacy of umbilical mesenchymal stem cell transplantation via renal subcapsular and parenchymal routes in AKI-CKD mice. Stem Cell. Res. Ther. 13 (1), 128. 10.1186/s13287-022-02805-3 35337372 PMC8953025

[B26] Huang Y. Q. Liang C. H. He L. Tian J. Liang C. S. Chen X. (2016). Development and validation of a radiomics nomogram for preoperative prediction of lymph node metastasis in colorectal cancer. J. Clin. Oncol. 34 (18), 2157–2164. 10.1200/jco.2015.65.9128 27138577

[B27] Jahaj E. Vassiliou A. G. Pratikaki M. Gallos P. Mastora Z. Dimopoulou I. (2021). Serum neutrophil gelatinase-associated lipocalin (NGAL) could provide better accuracy than creatinine in predicting acute kidney injury development in critically ill patients. J. Clin. Med. 10 (22), 5379. 10.3390/jcm10225379 34830657 PMC8625137

[B28] Ji W. Liu H. Liu C. Shao L. Liu Y. Fan S. (2017). Up-regulation of MCM3 relates to neuronal apoptosis after traumatic brain injury in adult rats. Cell. Mol. Neurobiol. 37 (4), 683–693. 10.1007/s10571-016-0404-x 27401074 PMC11482084

[B29] Jiang X. Stockwell B. R. Conrad M. (2021). Ferroptosis: Mechanisms, biology and role in disease. Nat. Rev. Mol. Cell. Biol. 22 (4), 266–282. 10.1038/s41580-020-00324-8 33495651 PMC8142022

[B30] Kam Tao Li P. Burdmann E. A. Mehta R. L. World Kidney Day Steering Committee 2013 (2013). Acute kidney injury: Global health alert. J. Nephropathol. 2 (2), 90–97. 10.12860/jnp.2013.15 24475433 PMC3891141

[B31] Kim J. Y. Bai Y. Jayne L. A. Abdulkader F. Gandhi M. Perreau T. (2020). SOX9 promotes stress-responsive transcription of VGF nerve growth factor inducible gene in renal tubular epithelial cells. J. Biol. Chem. 295 (48), 16328–16341. 10.1074/jbc.RA120.015110 32887795 PMC7705303

[B32] Koyner J. L. Vaidya V. S. Bennett M. R. Ma Q. Worcester E. Akhter S. A. (2010). Urinary biomarkers in the clinical prognosis and early detection of acute kidney injury. Clin. J. Am. Soc. Nephrol. 5 (12), 2154–2165. 10.2215/cjn.00740110 20798258 PMC2994075

[B33] Li X. Mu G. Song C. Zhou L. He L. Jin Q. (2018). Role of M2 macrophages in sepsis-induced acute kidney injury. SHOCK 50 (2), 233–239. 10.1097/shk.0000000000001006 28953574

[B34] Licciulli S. Cambiaghi V. Scafetta G. Gruszka A. M. Alcalay M. (2010). Pirin downregulation is a feature of AML and leads to impairment of terminal myeloid differentiation. LEUKEMIA 24 (2), 429–437. 10.1038/leu.2009.247 20010624

[B35] Linkermann A. Chen G. Dong G. Kunzendorf U. Krautwald S. Dong Z. (2014). Regulated cell death in AKI. J. Am. Soc. Nephrol. 25 (12), 2689–2701. 10.1681/asn.2014030262 24925726 PMC4243360

[B36] Liu Y. Li L. Jiang D. Yang M. Gao X. Lv K. (2021). A novel nomogram for survival prediction of patients with spinal metastasis from prostate cancer. SPINE 46 (6), E364–e373. 10.1097/brs.0000000000003888 33620180

[B37] Martin-Sanchez D. Ruiz-Andres O. Poveda J. Carrasco S. Cannata-Ortiz P. Sanchez-Niño M. D. (2017). Ferroptosis, but not necroptosis, is important in nephrotoxic folic acid-induced AKI. J. Am. Soc. Nephrol. 28 (1), 218–229. 10.1681/asn.2015121376 27352622 PMC5198282

[B38] Mehta R. L. Cerdá J. Burdmann E. A. Tonelli M. García-García G. Jha V. (2015). International society of nephrology's 0by25 initiative for acute kidney injury (zero preventable deaths by 2025): A human rights case for nephrology. Lancet 385 (9987), 2616–2643. 10.1016/s0140-6736(15)60126-x 25777661

[B39] Melo Ferreira R. Sabo A. R. Winfree S. Collins K. S. Janosevic D. Gulbronson C. J. (2021). Integration of spatial and single-cell transcriptomics localizes epithelial cell-immune cross-talk in kidney injury. JCI Insight 6 (12), e147703. 10.1172/jci.insight.147703 34003797 PMC8262485

[B40] Mishima E. Sato E. Ito J. Yamada K. I. Suzuki C. Oikawa Y. (2020). Drugs repurposed as antiferroptosis agents suppress organ damage, including AKI, by functioning as lipid peroxyl radical scavengers. J. Am. Soc. Nephrol. 31 (2), 280–296. 10.1681/asn.2019060570 31767624 PMC7003311

[B41] Newman A. M. Liu C. L. Green M. R. Gentles A. J. Feng W. Xu Y. (2015). Robust enumeration of cell subsets from tissue expression profiles. Nat. METHODS 12 (5), 453–457. 10.1038/nmeth.3337 25822800 PMC4739640

[B42] Orzaez D. de Jong A. J. Woltering E. J. (2001). A tomato homologue of the human protein PIRIN is induced during programmed cell death. PLANT Mol. Biol. 46 (4), 459–468. 10.1023/a:1010618515051 11485202

[B43] Reichling C. Taieb J. Derangere V. Klopfenstein Q. Le Malicot K. Gornet J. M. (2020). Artificial intelligence-guided tissue analysis combined with immune infiltrate assessment predicts stage III colon cancer outcomes in PETACC08 study. GUT 69 (4), 681–690. 10.1136/gutjnl-2019-319292 31780575 PMC7063404

[B44] Ritchie M. E. Phipson B. Wu D. Hu Y. Law C. W. Shi W. (2015). Limma powers differential expression analyses for RNA-sequencing and microarray studies. NUCLEIC ACIDS Res. 43 (7), e47. 10.1093/nar/gkv007 25605792 PMC4402510

[B45] Schmidt-Ott K. M. Mori K. Kalandadze A. Li J. Y. Paragas N. Nicholas T. (2006). Neutrophil gelatinase-associated lipocalin-mediated iron traffic in kidney epithelia. Curr. Opin. Nephrol. Hypertens. 15 (4), 442–449. 10.1097/01.mnh.0000232886.81142.58 16775460

[B46] Singbartl K. Formeck C. L. Kellum J. A. (2019). Kidney-immune system crosstalk in AKI. SEMINARS Nephrol. 39 (1), 96–106. 10.1016/j.semnephrol.2018.10.007 30606411

[B47] Söling A. Sackewitz M. Volkmar M. Schaarschmidt D. Jacob R. Holzhausen H. J. (2005). Minichromosome maintenance protein 3 elicits a cancer-restricted immune response in patients with brain malignancies and is a strong independent predictor of survival in patients with anaplastic astrocytoma. Clin. CANCER Res. 11 (1), 249–258. 10.1158/1078-0432.249.11.1 15671553

[B48] Stenson E. K. Kendrick J. Dixon B. Thurman J. M. (2023). The complement system in pediatric acute kidney injury. Pediatr. Nephrol. 38 (5), 1411–1425. 10.1007/s00467-022-05755-3 36203104 PMC9540254

[B49] van der Elst G. Varol H. Hermans M. Baan C. C. Duong-van Huyen J. P. Hesselink D. A. (2023). The mast cell: A janus in kidney transplants. Front. Immunol. 14, 1122409. 10.3389/fimmu.2023.1122409 36891297 PMC9986315

[B50] Wang Y. Quan F. Cao Q. Lin Y. Yue C. Bi R. (2021). Quercetin alleviates acute kidney injury by inhibiting ferroptosis. J. Adv. Res. 28, 231–243. 10.1016/j.jare.2020.07.007 33364059 PMC7753233

[B51] Wang Y. Zhang M. Bi R. Su Y. Quan F. Lin Y. (2022). ACSL4 deficiency confers protection against ferroptosis-mediated acute kidney injury. Redox Biol. 51, 102262. 10.1016/j.redox.2022.102262 35180475 PMC8857079

[B52] Xiao J. Yang Q. Zhang Y. Xu H. Ye Y. Li L. (2021). Maresin conjugates in tissue regeneration-1 suppresses ferroptosis in septic acute kidney injury. Cell. Biosci. 11 (1), 221. 10.1186/s13578-021-00734-x 34961563 PMC8711186

[B53] Xin S. Mueller C. Pfeiffer S. Kraft V. A. N. Merl-Pham J. Bao X. (2022). MS4A15 drives ferroptosis resistance through calcium-restricted lipid remodeling. Cell. DEATH Differ. 29 (3), 670–686. 10.1038/s41418-021-00883-z 34663908 PMC8901757

[B54] Yu G. Wang L. G. Han Y. He Q. Y. (2012). clusterProfiler: an R package for comparing biological themes among gene clusters. Omics 16 (5), 284–287. 10.1089/omi.2011.0118 22455463 PMC3339379

[B55] Zhang H. Liu X. Zhou L. Deng Z. Wang Y. (2022). Identification of RPS7 as the biomarker of ferroptosis in acute kidney injury. Biomed. Res. Int. 2022, 3667339. 10.1155/2022/3667339 36277893 PMC9584673

[B56] Zhao Z. Wu J. Xu H. Zhou C. Han B. Zhu H. (2020). XJB-5-131 inhibited ferroptosis in tubular epithelial cells after ischemia-reperfusion injury. Cell. Death Dis. 11 (8), 629. 10.1038/s41419-020-02871-6 32796819 PMC7429848

[B57] Zhou N. Yuan X. Du Q. Zhang Z. Shi X. Bao J. (2023). FerrDb V2: Update of the manually curated database of ferroptosis regulators and ferroptosis-disease associations. NUCLEIC ACIDS Res. 51 (D1), D571–d582. 10.1093/nar/gkac935 36305834 PMC9825716

[B58] Zhou Y. Shi W. Zhao D. Xiao S. Wang K. Wang J. (2022). Identification of immune-associated genes in diagnosing aortic valve calcification with metabolic syndrome by integrated bioinformatics analysis and machine learning. Front. Immunol. 13, 937886. 10.3389/fimmu.2022.937886 35865542 PMC9295723

[B59] Zou Y. Palte M. J. Deik A. A. Li H. Eaton J. K. Wang W. (2019). A GPX4-dependent cancer cell state underlies the clear-cell morphology and confers sensitivity to ferroptosis. Nat. Commun. 10 (1), 1617. 10.1038/s41467-019-09277-9 30962421 PMC6453886

